# Whole Blood Gene Expression Differentiates between Atrial Fibrillation and Sinus Rhythm after Cardioversion

**DOI:** 10.1371/journal.pone.0157550

**Published:** 2016-06-22

**Authors:** Kripa Raman, Stefanie Aeschbacher, Matthias Bossard, Thomas Hochgruber, Andreas J. Zimmermann, Beat A. Kaufmann, Katrin Pumpol, Peter Rickenbacker, Guillaume Paré, David Conen

**Affiliations:** 1 Population Health Research Institute, David Braley Cardiac, Vascular and Stroke Research Institute, 237 Barton Street East, Hamilton, ON, L8L 2X2, Canada; 2 Thrombosis and Atherosclerosis Research Institute, David Braley Cardiac, Vascular and Stroke Research Institute, 237 Barton Street East, Hamilton, ON, L8L 2X2, Canada; 3 Department of Medical Sciences, McMaster University, 1280 Main Street West, Hamilton, ON, L8S 4K1, Canada; 4 Division of Internal Medicine, Department of Medicine, University Hospital Basel, Petersgraben 4, 4031, Basel, Switzerland; 5 Cardiovascular Research Institute Basel, University Hospital Basel, Spitalstrasse 2, 4031, Basel, Switzerland; 6 Cardiology Division, Department of Medicine, University Hospital Basel, Petersgraben 4, 4031, Basel, Switzerland; 7 Division of Cardiology, Hamilton General Hospital, Hamilton Health Sciences, 237 Barton Street East, Hamilton, ON, L8L 2X2, Canada; 8 Cardiology Division, Kantonsspital Bruderholz, 4101, Bruderholz, Switzerland; 9 Department of Pathology and Molecular Medicine, McMaster University, Michael G. DeGroote School of Medicine, 1280 Main Street West, Hamilton, ON, L8S 4K1, Canada; University of Minnesota, UNITED STATES

## Abstract

**Background:**

Treatment to restore sinus rhythm among patients with atrial fibrillation (AF) has limited long-term success rates. Gene expression profiling may provide new insights into AF pathophysiology.

**Objective:**

To identify biomarkers and improve our understanding of AF pathophysiology by comparing whole blood gene expression before and after electrical cardioversion (ECV).

**Methods:**

In 46 patients with persistent AF that underwent ECV, whole blood samples were collected 1–2 hours before and 4 to 6 weeks after successful cardioversion. The paired samples were sent for microarray and plasma biomarker comparison.

**Results:**

Of 13,942 genes tested, expression of *SLC25A20* and *PDK4* had the strongest associations with AF. Post-cardioversion, *SLC25A20* and *PDK4* expression decreased by 0.8 (CI 0.7–0.8, *p* = 2.0x10^-6^) and 0.7 (CI 0.6–0.8, *p* = 3.0x10^-5^) fold respectively. Median N-terminal pro B-type natriuretic peptide (NT-proBNP) concentrations decreased from 127.7 pg/mL to 44.9 pg/mL (*p* = 2.3x10^-13^) after cardioversion. AF discrimination models combining NT-proBNP and gene expression (NT-proBNP + *SLC25A20* area under the curve = 0.88, NT-proBNP + *PDK4* AUC = 0.86) had greater discriminative capacity as compared with NT-proBNP alone (AUC = 0.82). Moreover, a model including NT-proBNP, *SLC25A20* and *PDK4* significantly improved AF discrimination as compared with other models (AUC = 0.87, Net Reclassification Index >0.56, *p*<5.8x10^-3^). We validated the association between *SLC25A20* and *PDK4* with AF in an independent sample of 17 patients.

**Conclusion:**

This study demonstrates that *SLC25A20*, *PDK4*, and NT-proBNP have incremental utility as biomarkers discriminating AF from sinus rhythm. Elevated *SLC25A20* and *PDK4* expression during AF indicates an important role for energy metabolism in AF.

## Introduction

Atrial fibrillation (AF), the most common cardiac arrhythmia, is associated with an increased risk of death, stroke [1,2] and heart failure [2–4]. Its incidence is projected to increase with the ageing of population and rising prevalence of obesity [5–7]. However, treatment strategies aiming to revert AF to sinus rhythm have limited long-term success rates and significant risks [8,9]. While it has been demonstrated that a substantial proportion of AF originates from the pulmonary veins [10], our current understanding of the complex pathophysiology remains incomplete. Improvements in this area may not only help to develop novel treatment strategies for AF patients, but also to anticipate the rhythm stability among patients with AF, and to develop better methods to detect intermittent forms of AF.

We hypothesized that identification of novel biomarkers associated with rhythm changes among AF patients may provide insights into our pathophysiologic understanding of the disease. Assessment of whole blood gene expression is an emerging and promising class of biomarkers. Gene expression levels vary rapidly in response to physiologic changes and may have disease specificity that outreaches conventional biomarkers. Recently, gene expression patterns of left atrial tissue from AF patients has been described [11–13]. In addition, the Framingham group has analyzed peripheral blood gene expression among patients with prevalent AF as compared with a large population of non-affected individuals [14]. However, neither of these studies has assessed changes in peripheral blood gene expression within an individual patient after conversion from AF to sinus rhythm.

We therefore aimed to study AF patients pre- and post-electrical cardioversion (ECV), in order to identify AF specific whole blood RNA biomarkers potentially implicated in AF pathophysiology. We also wanted to assess the discriminative capacity of RNA biomarkers. Finally, novel gene expression biomarkers were validated in an independent group of participants.

## Methods

### Study population

We prospectively enrolled patients >18 years with persistent AF, defined as a non-self terminating episode lasting >7 days, who were scheduled for non-urgent electrical cardioversion (ECV) at two tertiary hospitals in Switzerland. We excluded patients with untreated severe valvular disease, unstable and acute heart failure, limiting active or chronic major diseases, and a history of open-heart surgery within 3 months of inclusion. Informed written consent was obtained from all patients and the study was approved by Ethics Committee Basel Switzerland.

### Study procedures

Study visits were scheduled approximately 24 hours before electrical cardioversion and after 4 ± 1 weeks of follow-up. Information on baseline characteristics, concomitant medication and co-morbidities was collected through study questionnaires both at baseline and follow-up. In addition, conventional blood pressure measurements, standard 12-lead electrocardiogram (ECG), 24-hour Holter ECG monitoring, real time 3-dimensional echocardiography and blood sampling were obtained at both visits. At baseline, all examinations were performed 1–2 hours prior to the cardioversion procedure. ECV was performed according to local standards. After cardioversion, changes in personal medication were strongly discouraged until the follow-up visit. The second blood sampling was obtained directly after the follow-up 24-hour Holter ECG, in order to confirm stable sinus rhythm. Patients who had recurrent AF between the two scheduled visits were excluded from this study.

### Blood sampling and biomarker measurements

Prior to ECV and at follow-up, venous blood samples were collected in EDTA tubes and PAXgene™ Blood RNA tubes (PreAnalytiX). EDTA tubes were immediately centrifuged to isolate plasma and all tubes were stored at -80°C. High-sensitivity C-reactive protein (hs-CRP), cystatin C (CYSC), and interleukin-6 (IL6) were measured on a Beckman Coulter Unicel DxC600 Synchron Clinical System (Beckman) according to the manufacturer’s protocol. Myeloperoxidase (MPO) was measured using the ARCHITECT MPO immunoassay on the ARCHITECT Clinical Chemistry Analyzer (Abbott). N-terminal pro B-type natriuretic peptide (NT-proBNP) was measured on the Elecsys 2010 immunoassay analyzer (Roche).

### RNA extraction

The PAXgene™ Blood RNA tubes were processed at the Genetic and Molecular Epidemiology Laboratory of PHRI and McMaster University, Hamilton ON. Paired samples were processed using the same RNA extraction and amplification method. Total RNA was isolated from samples using the QIAsymphony PAXgene Blood RNA Kit (QIAGEN) or the MagMAX Stabilized Blood Tube RNA Isolation Kit (LifeTech). RNA was then quantified with RiboGreen® (LifeTech) and Nanodrop (Nanodrop).

### Microarray hybridization

200ng of total RNA was amplified and biotinylated according to the manufacturer’s protocol. Samples were amplified with the TotalPrep RNA Amplification Kit (LifeTech) or the lllumina TotalPrep-96 RNA Amplification Kit (LifeTech). The final biotin-labeled cRNA species were then hybridized to the Illumina Human HT-12v4 expression BeadChips (Illumina). Each BeadChip hold 12 samples at a time so paired samples were hybridized on to the same chip. BeadChips were then washed, dried and scanned on the iScan System (Illumina) as per the manufacturer’s protocol.

### Microarray pre-processing and quality control

The Illumina Human HT-12v4 BeadChip interrogates expression of 34,694 unique genes using 47,323 probes. The raw BeadChip sample probe profile and control probe profile were exported from GenomeStudio version 1.9.0 (Illumina). All data preprocessing and quality control was performed in R (http://r-project.org). Four samples were found to be outliers. The samples did not pass quality control metrics [15], as the average intensity was significantly different from other arrays. Thus the four samples and their corresponding pairs were excluded from further analysis. Data pre-processing involved background correction using the non-genomic control probes, quantile normalization and log2 transformation [16,17]. Probes with detection P-value <0.05 in >50% of the samples were included for further statistical analysis. The final pre-processed data included expression values for 13,942 RNA probes for each of the 92 samples from 46 individuals. Due to consent form restrictions, expression data cannot be made publicly available.

### Quantitative Real-time Polymerase Chain Reaction

Reverse transcription was performed using the QuantiTect Reverse Transcription Kit (Qiagen). *SLC25A20* expression was monitored with the Hs00386383_m1 probe (LifeTech), *PDK4* with the Hs01037712_m1 probe (LifeTech) and *ITGB5* with the Hs00174435_m1 probe (LifeTech) as per the manufacturer protocol. Each qPCR was performed in duplex with the housekeeping gene *ACTB*, measured using the Hs01060665_g1 probe (LifeTech), to normalize expression. The TaqMan qPCR was conducted on a Viia7 Real-Time System (LifeTech) and cycle threshold (CT) values were calculated automatically with default parameters. Fold change (FC) differences were calculated using the **δ**CT method.

### Statistical analysis

All statistical analyses were performed using R. Clinical demographics were grouped according to pre- or post-cardioversion status. Normally distributed variables were compared using paired Student T-tests; otherwise paired Wilcoxon rank sum tests were used. A two-sided p-value<0.05 was considered statistically significant.

Microarrays (and quantitative PCR) measure relative rather than absolute gene expression, or in other words the relative increase or decrease in expression of a gene as compared with global expression (or housekeeping genes). Differential gene expression was thus reported as FC, with 95% confidence intervals (CI). Linear regression models were used to identify differentially expressed RNA transcripts after cardioversion (during sinus rhythm) as compared with pre-cardioversion samples (during AF). Each model tested a single gene’s association with AF while adjusting for sample pairs. To correct for multiple hypothesis testing a conservative Bonferroni correction was applied, setting the significance threshold at 0.05 / 13,942 = 3.6 x 10^−6^.

Significant genes and plasma biomarkers were also tested for association with AF risk factors (gender, age, BMI, systolic blood pressure and diastolic blood pressure), Holter ECG (heart rate, heart rate maximum and minimum) and echocardiography (E wave, A wave, deceleration time, left atrial volume, left atrial maximum volume, left ventricular ejection fraction) parameters using linear regression models. An adjusted p-value <0.05/14 = 0.0036 was considered significant. Paired Wilcoxon rank sum test was used to compare biomarker levels pre- and post-cardioversion. Receiver operating characteristic (ROC) curves were constructed, using pROC [18], to determine the discriminative capacity of significant biomarkers for AF status. The area under the ROC curve (AUC) was determined as a measure of sensitivity and specificity. To compare models we calculated the continuous Net Reclassification Index (NRI) [19] using Hmisc [20]. We considered an NRI greater than 0.6 a strong, 0.4 an intermediate, and 0.2 a weak improvement in discriminative capacity. To verify and validate microarray expression results qPCR data was analyzed with linear regression models and adjusted for sample pairs.

## Results

### Patient characteristics

Between March 2010 to April 2013, 108 consecutive patients with persistent AF were enrolled into the study. 67 patients had successful cardioversion and confirmed sinus rhythm at the follow-up visit; 50 were selected for biomarker discovery and 17 for independent validation. After microarray quality control, the biomarker discovery cohort was reduced to 46 patients. Patient demographics in the discovery cohort are presented in Table 1. Mean age was 65.4 ± 10.6 and 26.1% of participants were female. The post-cardioversion follow-up examination took place 35 days ± 8 days after ECV and there were no significant changes in medication (p>0.05 = NS). Successful cardioversion resulted in a significant decrease in heart rate (pre 87 ± 17 vs. post 60 ± 10, p = 8.7x10^-14^) and a non-significant decrease in left atrial maximum volume (pre 91.7mL, post 72.9, p = 0.022). We also observed a decrease in E-wave velocity (pre 0.93 m/s, post 0.77 m/s, p = 2.8x10^-4^), an increase in deceleration time (pre 173 ms, post 243 ms, p = 1.0x10^-3^) and an increase in left ventricular ejection fraction (pre 44.3%, post 54.8%, p = 1.3x10^-3^) following cardioversion.

**Table 1:** Participant demographics for biomarker discovery cohort.

	Pre-cardioversion(n = 46)	Post-cardioversion (n = 46)	P-value
**Gender, n (% female)**	12 (26.1)	12 (26.1)	
**Age (years), mean ± SD**	65.4 ± 11.0	65.5 ± 11.0	
**BMI (kg/m**^**2**^**), mean ± SD**	27.6 ± 3.7	27.7 ± 3.7	
**Systolic BP (mmHg), mean ± SD**	136.3 ± 19.3	135.4 ± 18.6	0.71
**Diastolic BP (mmHg) mean ± SD**	82.3 ± 21.4	77.6 ± 9.5	0.067
**Diabetes, n (%)**	3 (6.5)	3 (6.5)	
**Medication, n(%)**			
**Aspirin**	2 (4.3)	3 (6.5)	1
**Vitmain K Antagonist**	46 (100)	42 (91.3)	0.07
**Beta-blocker**	37 (80.4)	33 (71.7)	0.07
**Class III Antiarrhythmic**	21 (45.7)	20 (43.5)	0.80
**Class Ic Antiarrhythmic**	1 (2.2)	2 (4.3)	1
**ACE Inhibitor**	28 (60.9)	27 (58.7)	0.77
**Diuretics**	22 (47.8)	18 (39.1)	0.13
**Calcium Antagonist**	7 (15.2)	5 (10.9)	0.42
**Digoxin**	3 (6.5)	1 (2.2)	0.35
**Aldosterone Antagonist**	2 (4.3)	3 (6.5)	1
**Statin**	18 (39.1)	15 (32.6)	0.15
**Holter ECG, mean ± SD**			
**Heart rate (bpm)**	87 ± 17	60 ± 10	8.7 x 10^−14^
**HR max (bpm)**	163 ± 36	104 ± 22	2.1 x10^-14^
**HR min (bpm)**	43 ± 9	42 ± 7	0.41
**Echocardiography, median (IQR)**			
**E wave (m/s)**	0.93 (0.8–1.0)	0.77 (0.7–0.9)	2.8 x10^-4^
**A wave (m/s)**	NA	0.58 (0.4–0.8)	NA
**E wave deceleration time (ms)**	173 (152.5–216.8)	243 (197–297)	1.0 x 10^−3^
**LA max volume (mL)**	91.7 (75.8–102.4)	72.9 (63.7–89.2)	0.022
**LV ejection fraction (%)**	44.3 (38.0–55.1)	54.8 (48.1–58.2)	1.3 x 10^−3^

### Association between gene expression and AF

**Fig 1:**
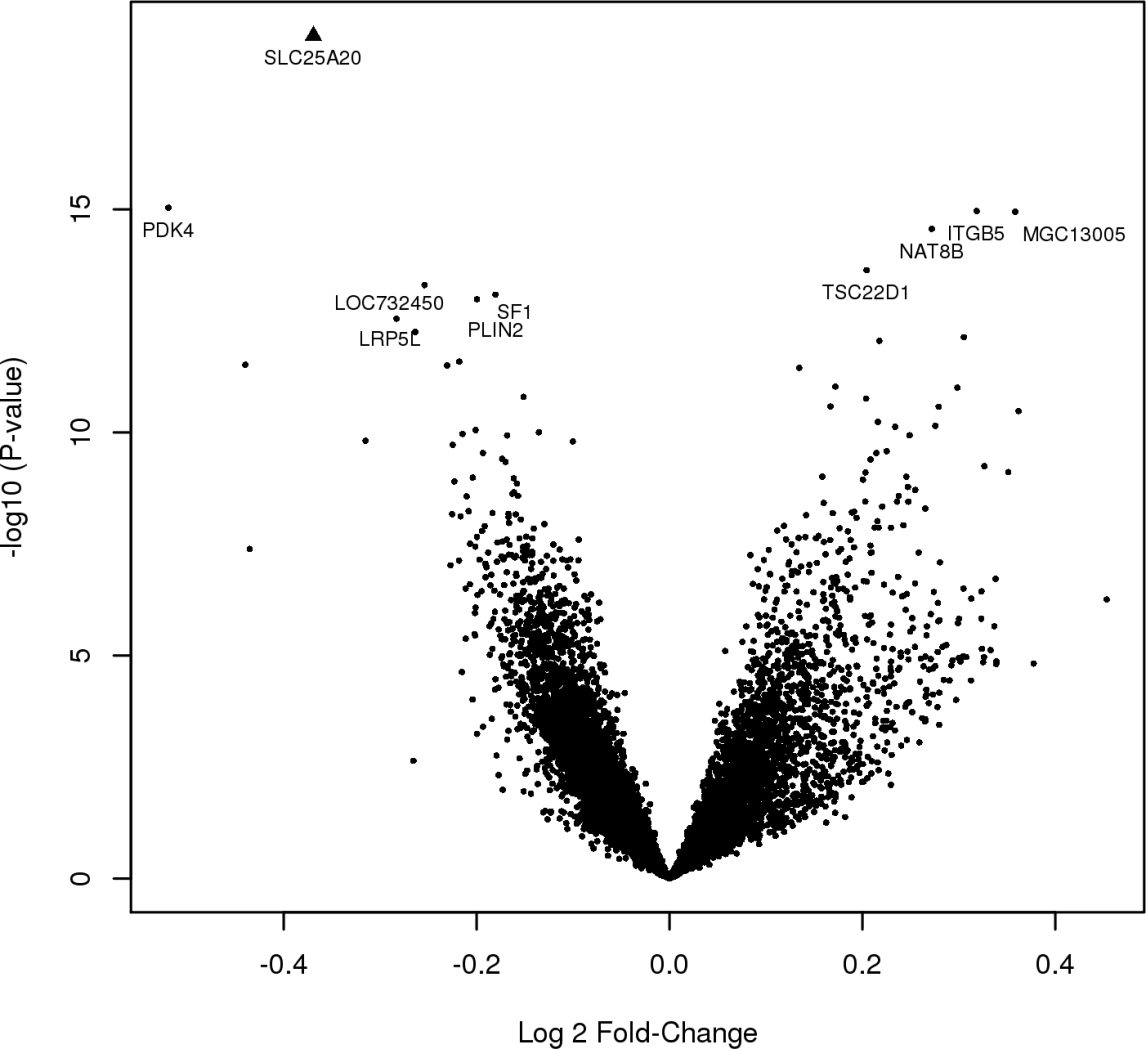
Volcano plot of gene expression changes pre- and post-cardioversion. Each point represents one of the RNA transcripts tested and the ten most significant genes have been labeled. The x-axis represents the effect of each gene, reported as log2 fold change, and a positive log2 fold change is indicative of increased expression in post-cardioversion samples. The y-axis represents the–log10(P-value). Triangle points represent genes that have significant differential expressed after Bonferroni correction (P-value <3.6x10^-6^).

Each of the 13,942 RNA probes was tested for association with AF while adjusting for sample pairs. The ten most significant genes from the analysis are presented in Table 2. *SLC25A20* expression was most significantly associated with AF, and the only gene that remained significant after adjustment for multiple hypothesis testing (Fig 1). A 0.8 fold decrease in *SLC25A20* was observed in post-cardioversion samples as compared with baseline (CI 0.7–0.8, p = 2.0x10^-6^, S1 Fig). *PDK4* was the second most significant gene and decreased by 0.7 fold post-cardioversion (CI 0.6–0.8, p = 3.0x10^-5^, S2 Fig). *ITGB5* was the third most significant gene and increased by 1.2 fold post-cardioversion (CI 1.1–1.4, p = 3.1x10^-5^, S3 Fig).

**Table 2:** Top genes associated with stable sinus rhythm after electrical cardioversion. Fold Change (FC). Confidence Interval (CI).

Gene	P-value	FC	Upper CI	Lower CI	Description
*SLC25A20*	2.0 x10^-6^	0.8	0.8	0.7	Solute carrier family 25 (carnitine/acylcarnitine translocase), member 20
*PDK4*	3.0 x10^-5^	0.7	0.8	0.6	Pyruvate dehydrogenase kinase, isozyme 4
*ITGB5*	3.1 x10^-5^	1.2	1.4	1.1	Integrin, beta 5
*DDX11L2*	3.2 x10^-5^	1.3	1.4	1.2	DEAD/H box helicase 11
*NAT8B*	4.1 x10^-5^	1.2	1.3	1.1	N-acetyltransferase 8B
*TSC22D1*	7.8 x10^-5^	1.2	1.2	1.1	TSC22 domain family, member 1
*LOC732450*	9.9 x10^-5^	0.8	0.9	0.8	
*SF1*	1.1 x10^-4^	0.9	0.9	0.8	Splicing factor 1
*PLIN2*	1.2 x10^-4^	0.9	0.9	0.8	Perilipin 2
*LRP5L*	1.7 x10^-4^	0.8	0.9	0.7	Low density lipoprotein receptor-related protein

Differential expression of the top three genes, *SLC25A20*, *PDK4* and *ITGB5*, was verified using qPCR. A consistent decrease in *SLC25A20* expression was observed in post-cardioversion sinus rhythm samples as compared with baseline AF using qPCR (FC = 0.7, CI 0.6–0.7, p = 1.6x10^-9^, S1 Fig). Similarly, a 0.6 fold decrease in *PDK4* expression was observed post-cardioversion (CI 0.5–0.7, p = 4.0x10^-5^, S2 Fig). Differential expression of *ITGB5* could not be verified (*p* = 0.086, S3 Fig).

### *SLC25A20* and *PDK4* expression are not associated with clinical variables

Restricting the analysis to pre-cardioversion AF samples, we tested *SLC25A20* and *PDK4* for association with AF risk factors, Holter ECG and echocardiography parameters. We observed no association between pre-cardioversion expression of either gene and measured variables (all p>0.05 = NS). We also restricted the analysis to samples collected post-ECV. After correction for multiple hypotheses testing, we observed a modest association between post-cardioversion *SLC25A20* expression and elevated diastolic blood pressure (p = 0.0017). *PDK4* had no association with the measured variables.

### Association between plasma biomarkers and AF

NT-proBNP levels were significantly decreased in post-cardioversion samples, as compared with baseline (median 127.7 vs. 44.9 pg/mL, p = 2.3x10^-13^). Circulating levels of hs-CRP, CYSC, IL6, and MPO did not change between pre and post-cardioversion samples *(*Table 3*)*. Limiting the analysis to pre-cardioversion or post-cardioversion AF samples, we observed no association between NT-proBNP concentrations and AF risk factors, Holter ECG or echocardiography parameters (all p>0.5 = NS).

**Table 3:** Plasma biomarker concentrations in participants pre- and post-cardioversion. Data are medians (interquartile range).

	Pre-cardioversion n = 46	Post-cardioversion n = 46	P-Value
hs-CRP (mg/L)	1.7 (1.0–3.2)	2.2 (1.0–3.6)	0.39
CYSC (mg/L)	0.93 (0.8–1.0)	0.93 (0.8–1.0)	0.63
IL6 (pg/mL)	2.3 (1.9–3.5)	2.7 (1.9–3.5)	0.55
MPO (pmol/L)	1113 (541.3–1556)	1091 (704.2–1901)	0.10
NT-proBNP (pg/mL)	127.7 (82.9–210.2)	44.9 (19.9–80.4)	2.3x10^-13^

### Discriminative capacity of NT-proBNP, *SLC25A20* and *PDK4* for AF

A multivariable logistic regression model for AF including NT-proBNP, *SLC25A20* and *PDK4*, indicated that all three biomarkers were independently associated with AF *(*S1 Table*)*. The association remained significant after adjustment for left atrial maximum volume (S2 Table). To determine the discriminative capacity of each biomarker we constructed receiver operator characteristics (ROC) curves. In single variable models, the area under the ROC curve discriminating between pre-cardioversion and post-cardioversion samples was 0.82 (CI 0.74–0.91) for NT-proBNP, 0.78 (CI 0.68–0.88) for *SLC25A20* and 0.69 (CI 0.58–0.80) for *PDK4* (S4 Fig). A two variable model including NT-proBNP and expression of either gene strongly improved discrimination as compared with NT-proBNP alone (NT-proBNP + *SLC25A20* AUC = 0.86, CI 0.79–0.94, NRI = 0.65, p = 1.1x10^-3^; NT-proBNP + *PDK4* AUC = 0.84, CI 0.76–0.92, NRI = 0.61, p = 2.5x10^-3^). Moreover a model including all three biomarkers had the greatest discriminative capacity (AUC = 0.87, CI 0.80–0.95). The combination of NT-proBNP, *SLC25A20* and *PDK4* improved discrimination as compared with two variable models (all vs NT-proBNP+*SLC25A20* NRI = 0.61, p = 2.6x10^-3^; all vs NT-proBNP+*PDK4* NRI = 0.56, p = 5.8x10^-3^).

### Replication of *SLC25A20* and *PDK4* in the validation cohort

We validated the decrease in *SLC25A20* and *PDK4* expression post-cardioversion in an independent sample consisting of 17 individuals. Power to identify an association of similar effect size was estimated at 89% for *SLC25A20* and 65% for *PDK4*, using an alpha of 0.05/2 = 0.025. Patient demographics are shown in S3 Table. After successful cardioversion we observed decreased heart rate (pre = 82.8 bpm, post = 57.45 bpm, p = 1.3x10^-5^) and decreased diastolic blood pressure (pre = 84.4, post = 76.5, p = 0.029) similar to the discovery sample. We also detected a 0.8 fold decrease in *SLC25A20* (CI 0.7–0.9, p = 2.0x10^-4^, S5 Fig) and 0.7 fold decrease in *PDK4* (CI 0.5–1.0, p = 0.05, S5 Fig) in post-cardioversion sinus rhythm samples as compared with baseline AF. Restricting the analysis to pre-cardioversion AF samples or post-cardioversion sinus rhythm, we tested both *SLC25A20* and *PDK4* for association with AF risk factors and observed no association (*p* = NS for all comparisons).

## Discussion

The present study evaluated peripheral blood gene expression and plasma protein biomarkers associated with AF rhythm by comparing paired patient samples pre- and post-ECV. We identified novel associations between whole blood gene expression of *SCL25A20* and *PDK4* with AF. Expression of both genes was elevated in AF as compared with post-ECV sinus rhythm. Adding either RNA marker to a model with NT-proBNP strongly improved AF discrimination. A model including *SLC25A20*, *PDK4* and NT-proBNP had the greatest ability to discriminate between AF and sinus rhythm. The association between both *SLC25A20* and *PDK4* with rhythm status was confirmed in an independent validation cohort.

Our results demonstrate that RNA biomarkers can provide independent discriminative information to NT-proBNP. Multiple studies are currently evaluating the clinical utility of NT-proBNP as a marker for cardiac impairment. Such a biomarker may facilitate diagnosis of paroxysmal AF or reclassification of cryptogenic stroke patients. Biomarker panels including NT-proBNP and RNA biomarkers may improve the specificity and sensitivity to detect cardiac dysrhythmias. Rapid point-of-care RNA tests are currently being developed [21], so peripheral blood RNA may potentially be integrated in routine clinical testing in the future.

Using transcriptome-wide expression profiling we identified an association between AF and elevated *SLC25A20* expression. *SLC25A20* encodes the carnitine-acylcarnitine translocase (CACT), which transports fatty acids into the inner mitochondrial membrane for β-oxidation [22,23]. We also observed increased expression of *PDK4* during AF prior to cardioversion. *PDK4* encodes pyruvate dehydrogenase kinase lipoamide kinase isozyme 4, which regulates the pyruvate dehydrogenase complex (PDC) [24]. PDC plays a critical role in glucose metabolism, converting pyruvate into acetyl-CoA for the citric acid cycle. Elevated expression of PDK4 inactivates PDC and promotes gluconeogenesis. Thus PDK4 expression is also of interest in diabetes, since its up-regulation can contribute to hyperglycemia [25]. Taken together, the observed decrease in *SLC25A20* and *PDK4* following cardioversion suggests an adaptive gene expression change in response to the metabolic demands of the heart. Thus, *SLC25A20* and *PDK4* expression may be associated with AF burden. In this context, recent studies showing that weight reduction was associated with reduced AF burden may also point towards the importance of energy metabolism in the occurrence of AF episodes [26,27].

In support of our results, a recent gene expression study observed decreased expression of *SLC25A20* in atrial tissue of patients that had no history of AF as compared with patients that had AF [11]. The researchers also detected a decrease in *SLC25A20* and *PDK4* in patients currently in sinus rhythm that had a history of AF, as compared with patients currently in AF. Our study confirms the potential importance of these RNA markers in the pathophysiology of AF. In addition, we show that expression changes can be observed not only across different patients, but also in an individual patient, if a sustained change in cardiac rhythm occurs. Considering the potential clinical applicability of these markers, it is of crucial importance that our study detected these expression changes in peripheral blood samples, given that atrial biopsies are not feasible in clinical practice.

The Framingham whole blood expression study [14] did not report an association between AF and *SLC25A20* or *PDK4*. These potential differences are not surprising since our study evaluated expression changes occurring during AF episodes as compared to sinus rhythm within the same individual, while the Framingham study assessed differential expression between individuals with and without AF.

There are limitations of our study, which need to be taken into account. First, we included only patients with persistent AF, and therefore generalizability to other AF populations remains uncertain. Second, all participants were of European origin thus the generalizability to other ethnicities remains uncertain. Third, fasting may have impacted gene expression. Pre-cardioversion samples were mostly collected after several hours of fasting, whereas fasting was not specified prior to post-cardioversion sampling. Studies have shown that free fatty acid concentrations increase with long term fasting [28,29]. Expression profiling of PBMCs after 24-hours of fasting has revealed increased expression of genes involved in fatty acid metabolism, including *SLC25A20* and *PDK4* [29]. However, studies have not described the time-course of expression changes with respect to the duration of fasting. As such the impact of shorter fasting episodes on gene expression, as in our study, has yet to be published. We have evaluated expression of *SLC25A20* and *PDK4* in a control population [30] for up to 12 hours of fasting and observed no association between expression and hours since last meal (p>0.5 = *NS*, S6 Fig). The significance of *SLC25A20* and *PDK4* in AF is further supported by the atrial tissue study that observed elevated expression of both genes in AF patients as compared with patients in sinus rhythm that had a history of AF [11]. Since surgery is required to collect atrial tissue, all individuals were fasting prior to sampling. Therefore, the results indicate that *SLC25A20* and *PDK4* are truly associated with AF. Finally, our study populations were relatively small, which may have hindered the detection of subtle gene expression differences during biomarker discovery. In addition, power calculations indicate that a larger sample size is required to validate the significance of *PDK4*.

In conclusion, the results of this study demonstrate that expression of *SLC25A20* and *PDK4* are independently associated with rhythm status among patients with persistent AF. These findings indicate that alterations in metabolic pathways are associated with the prevalent cardiac rhythm in an individual AF patient, providing not only novel pathophysiological insights but also new potential intervention targets that can be tested in future studies. In addition, our study demonstrates that NT-proBNP, *SLC25A20* and *PDK4* have incremental utility as biomarkers discriminating AF from sinus rhythm. Future studies should explore whether these markers may be helpful for predicting AF recurrence in clinical practice.

## Supporting Information

S1 Fig Boxplots of *SLC25A20* expression pre- and post- cardioversion. Boxes extend from the 25^th^ to the 75^th^ percentile, with the horizontal line representing the median. Outliers are identified as samples with an expression value 1.5 times more or less than the interquartile range. The CT (cycle threshold) is the number of PCR cycles required for the fluorescent signal to exceed background levels. Unlike microarray values, CT values are inversely proportional to the amount of target nucleic acid in a sample. A) Microarray expression of *SLC25A20* decreased following cardioversion. B) qPCR expression of *SLC25A20* also decreased following cardioversion. A symbol directly above a bar indicates a significant difference between groups; *p* <0.0005 (***).
(TIFF)

S2 Fig Boxplots of *PDK4* expression pre- and post-cardioversion. Boxes extend from the 25^th^ to the 75^th^ percentile, with the horizontal line representing the median. Outliers are identified as samples with an expression value 1.5 times more or less than the interquartile range. The CT (cycle threshold) is the number of PCR cycles required for the fluorescent signal to exceed background levels. Unlike microarray values, CT values are inversely proportional to the amount of target nucleic acid in a sample. A) Microarray expression of *PDK4* decreased following cardioversion. B) qPCR expression of *PDK4* also decreased following cardioversion. A symbol directly above a bar indicates a significant difference between groups; *p* <0.0005 (***).
(TIFF)

S3 Fig Boxplots of *ITGB5* expression pre- and post-cardioversion. Boxes extend from the 25^th^ to the 75^th^ percentile, with the horizontal line representing the median. Outliers are identified as samples with an expression value 1.5 times more or less than the interquartile range. The CT (cycle threshold) is the number of PCR cycles required for the fluorescent signal to exceed background levels. Unlike microarray values, CT values are inversely proportional to the amount of target nucleic acid in a sample. A) Microarray expression of *ITGB5* decreased following cardioversion. B) qPCR expression of *ITGB5* also decreased following cardioversion. A symbol directly above a bar indicates a significant difference between groups; *p* <0.0005 (***).
(TIFF)

S4 Fig Receiver-operating characteristic curves for the discrimination of pre-cardioversion AF from post-cardioversion sinus rhythm. (TIFF)

S5 Fig qPCR gene expression boxplots in the independent validation cohort. Boxes extend from the 25^th^ to the 75^th^ percentile, with the horizontal line representing the median. Outliers are identified as samples with an expression value 1.5 times more or less than the interquartile range. The CT (cycle threshold) is the number of PCR cycles required for the fluorescent signal to exceed background levels. Unlike microarray values, CT values are inversely proportional to the amount of target nucleic acid in a sample. A) qPCR expression of *SLC25A20* pre- and post- cardioversion in the independent validation cohort. B) qPCR expression of *PDK4*. A symbol directly above a bar indicates a significant difference between groups; *p* <0.0005 (***), *p* <0.05(*).
(TIFF)

S6 Fig Boxplots of microarray gene expression in control[30] population grouped based on hours from symptom onset. Boxes extend from the 25^th^ to the 75^th^ percentile, with the horizontal line representing the median. Outliers are identified as samples with an expression value 1.5 times more or less than the interquartile range. A) Expression of *SLC25A20*. B) Expression of *PDK4*.
(TIFF)

S1 Table Results of logistic regression model for AF status including biomarkers. A logistic regression model was constructed for rhythm status. The model included only NT-proBNP, *SLC25A20* and *PDK4*.
(DOCX)

S2 Table Results of logistic regression model for AF status including biomarkers and LA maximum volume. A logistic regression model was constructed for rhythm status. The model included NT-proBNP, *SLC25A20*, *PDK4* and left atrial maximum volume.
(DOCX)

S3 Table Demographics for the independent validation in participants with successful cardioversion. (DOCX)
