# Supplementary figures and images for: Whole Blood Gene Expression Differentiates between Atrial Fibrillation and Sinus Rhythm after Cardioversion
Source: PLoS One. 2016 Jun 22;11(6):e0157550. doi: 10.1371/journal.pone.0157550 (PMC4917233; doi:10.1371/journal.pone.0157550)

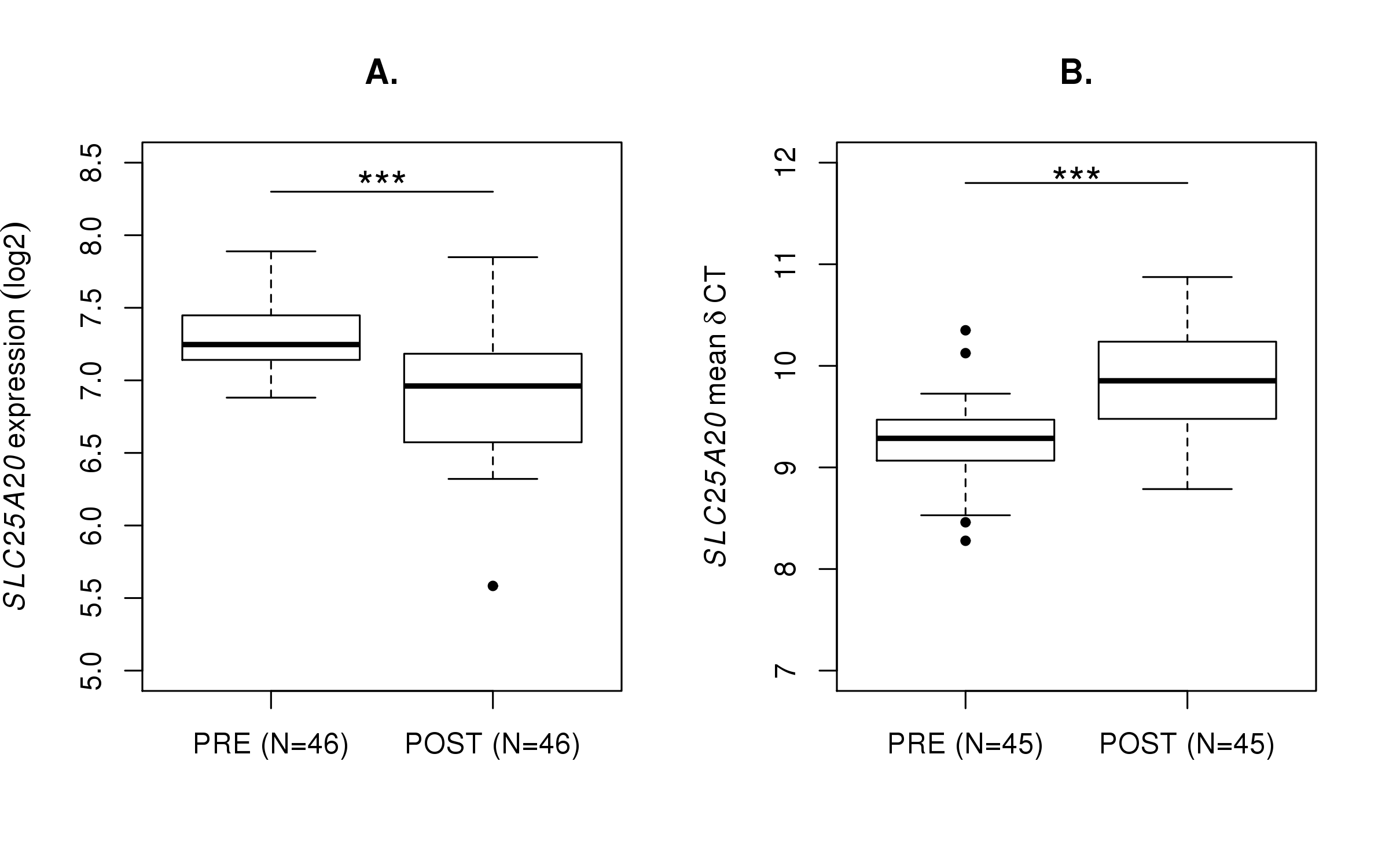

Supplement: S1 Fig — Boxes extend from the 25th to the 75th percentile, with the horizontal line representing the median. Outliers are identified as samples with an expression value 1.5 times more or less than the interquartile range. The CT (cycle threshold) is the number of PCR cycles required for the fluorescent signal to exceed background levels. Unlike microarray values, CT values are inversely proportional to the amount of target nucleic acid in a sample. A) Microarray expression of SLC25A20 decreased following cardioversion. B) qPCR expression of SLC25A20 also decreased following cardioversion. A symbol directly above a bar indicates a significant difference between groups; p <0.0005 (***). (TIFF) [file pone.0157550.s001.tiff]

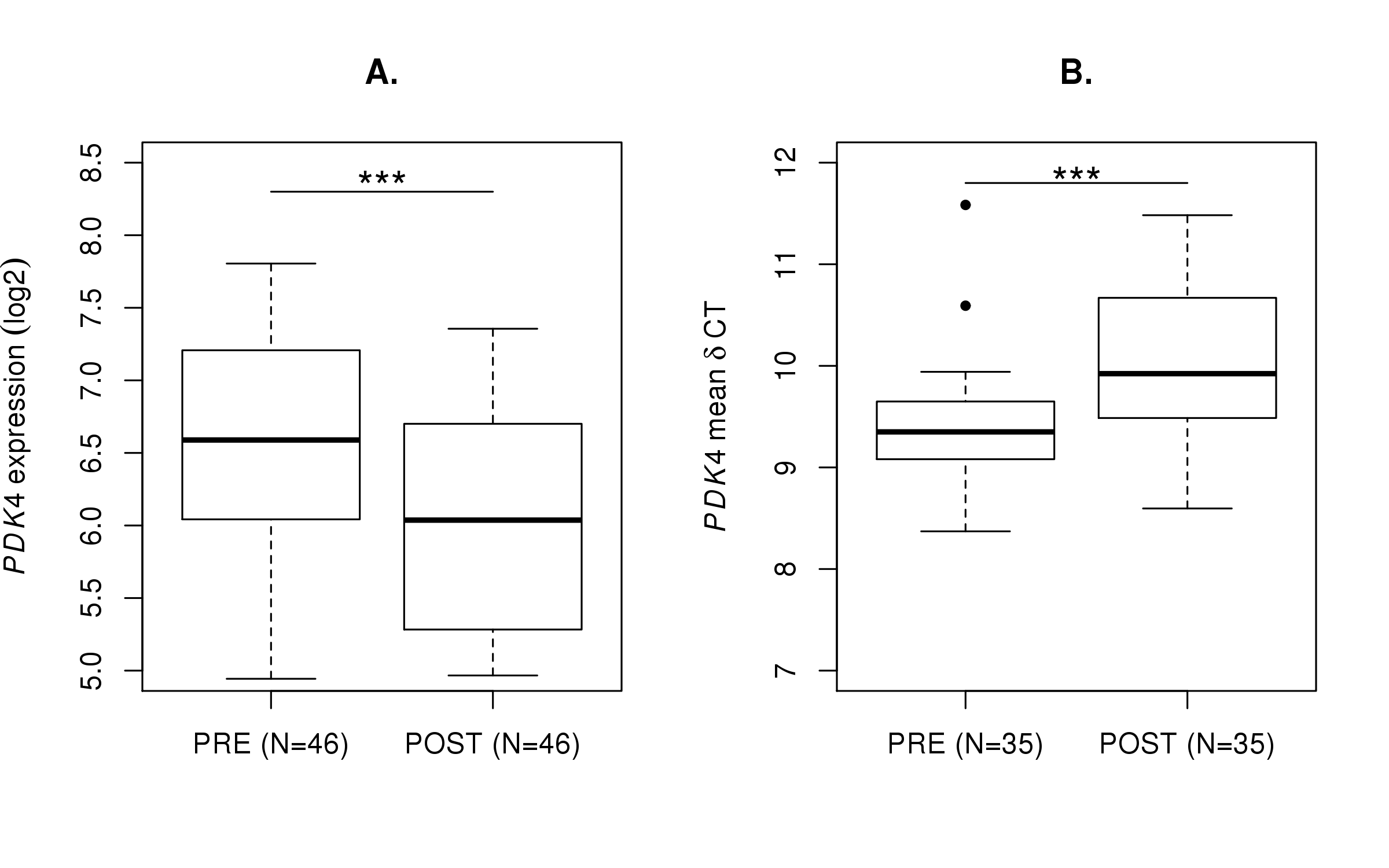

Supplement: S2 Fig — Boxes extend from the 25th to the 75th percentile, with the horizontal line representing the median. Outliers are identified as samples with an expression value 1.5 times more or less than the interquartile range. The CT (cycle threshold) is the number of PCR cycles required for the fluorescent signal to exceed background levels. Unlike microarray values, CT values are inversely proportional to the amount of target nucleic acid in a sample. A) Microarray expression of PDK4 decreased following cardioversion. B) qPCR expression of PDK4 also decreased following cardioversion. A symbol directly above a bar indicates a significant difference between groups; p <0.0005 (***). (TIFF) [file pone.0157550.s002.tiff]

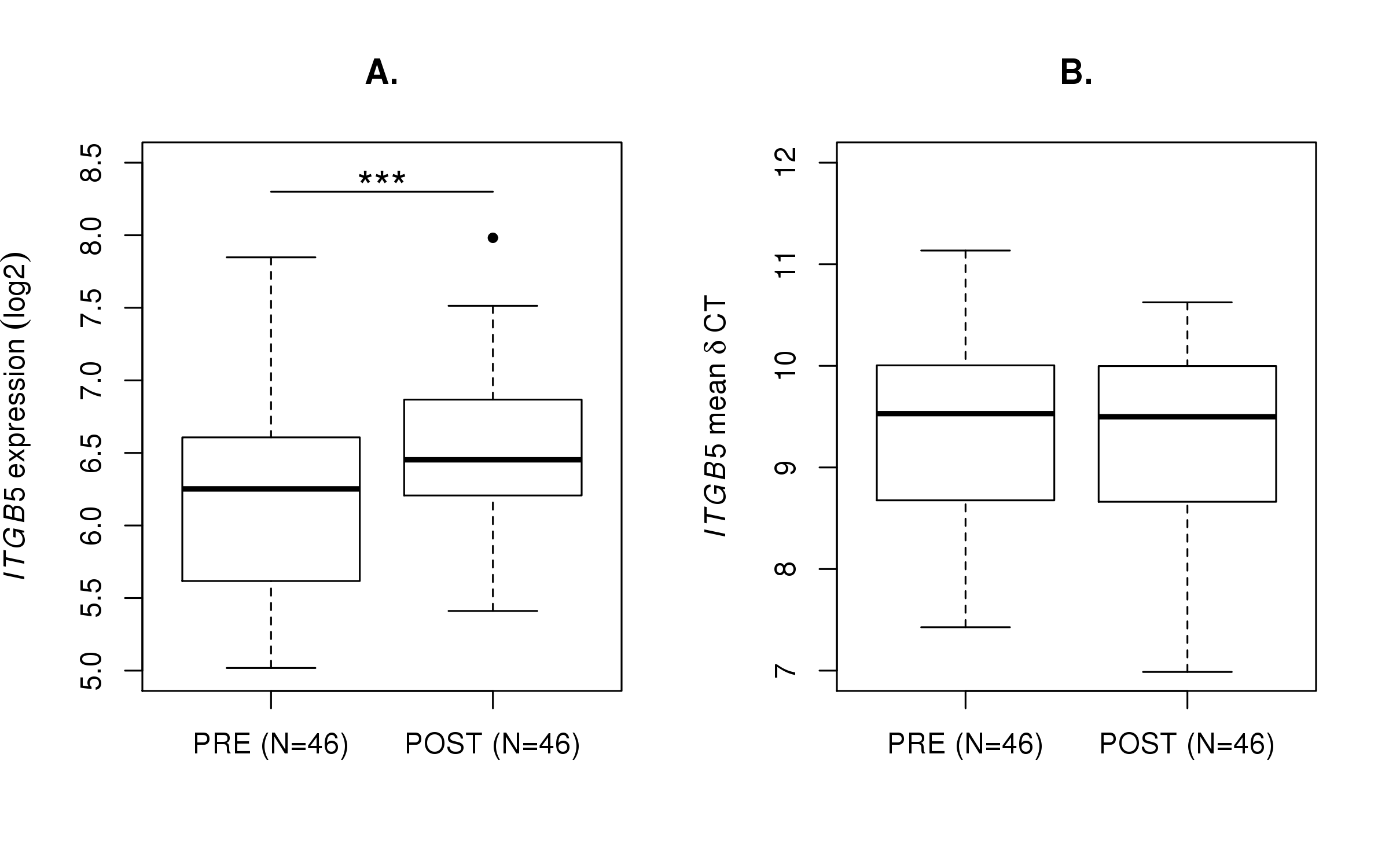

Supplement: S3 Fig — Boxes extend from the 25th to the 75th percentile, with the horizontal line representing the median. Outliers are identified as samples with an expression value 1.5 times more or less than the interquartile range. The CT (cycle threshold) is the number of PCR cycles required for the fluorescent signal to exceed background levels. Unlike microarray values, CT values are inversely proportional to the amount of target nucleic acid in a sample. A) Microarray expression of ITGB5 decreased following cardioversion. B) qPCR expression of ITGB5 also decreased following cardioversion. A symbol directly above a bar indicates a significant difference between groups; p <0.0005 (***). (TIFF) [file pone.0157550.s003.tiff]

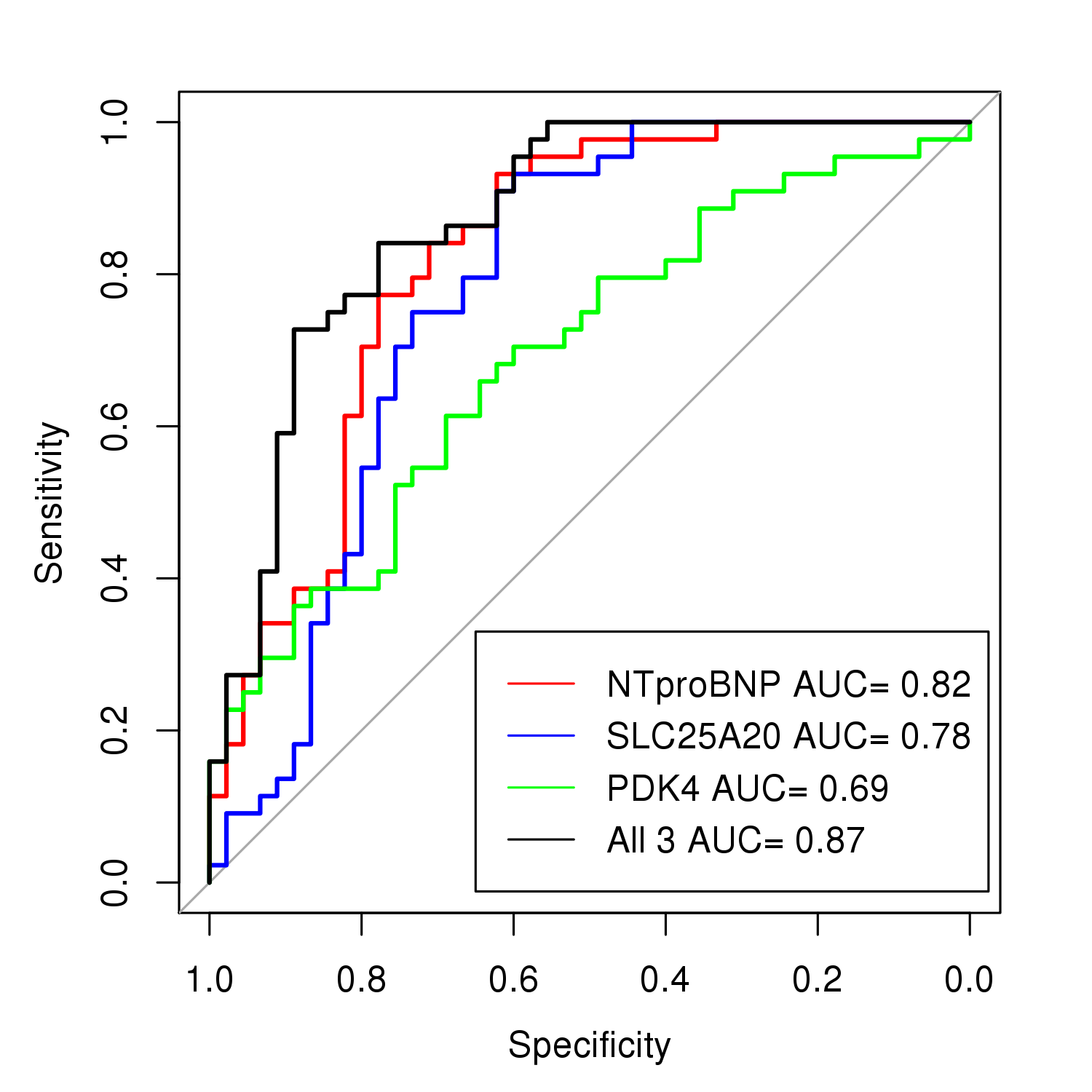

Supplement: S4 Fig — (TIFF) [file pone.0157550.s004.tiff]

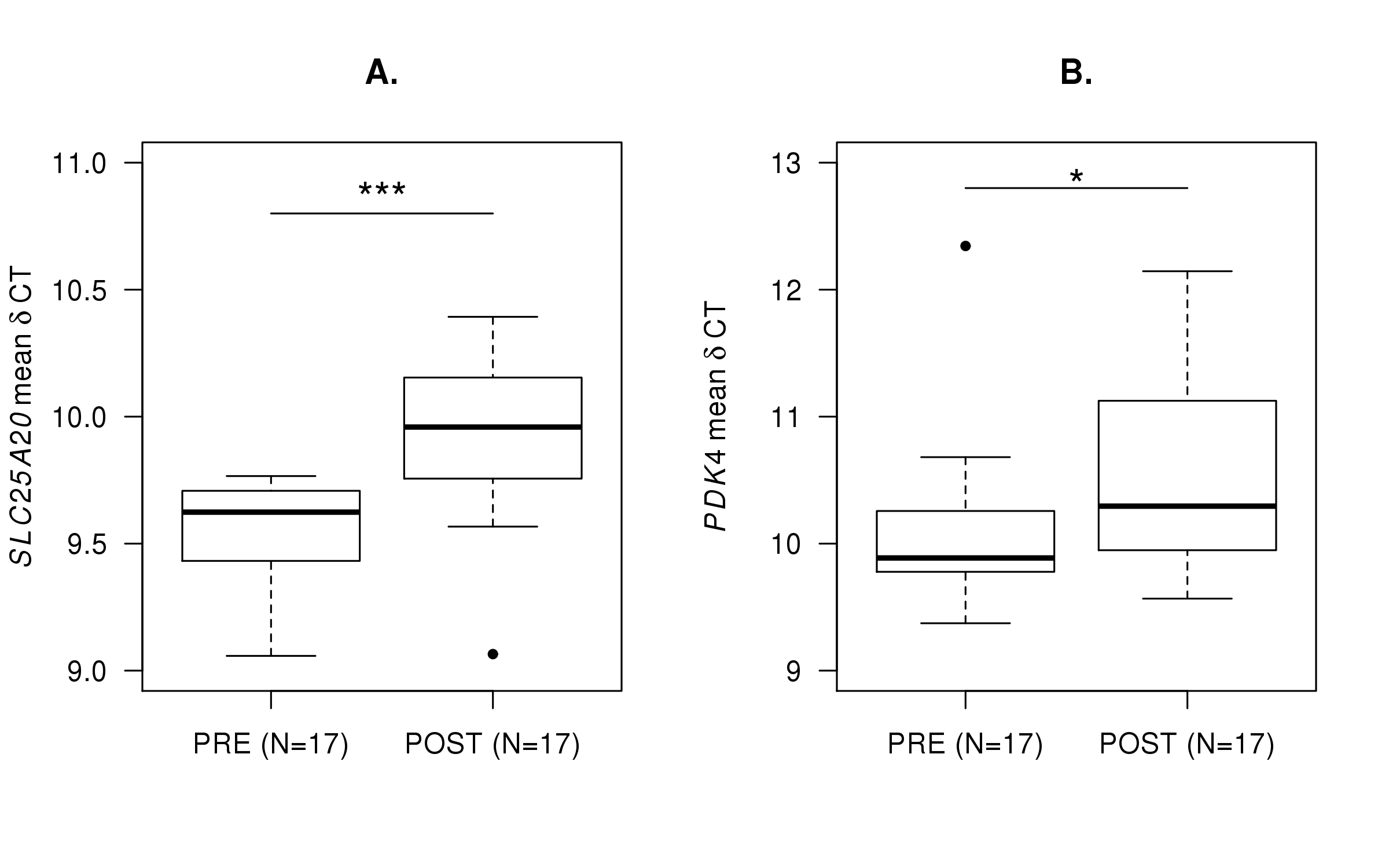

Supplement: S5 Fig — Boxes extend from the 25th to the 75th percentile, with the horizontal line representing the median. Outliers are identified as samples with an expression value 1.5 times more or less than the interquartile range. The CT (cycle threshold) is the number of PCR cycles required for the fluorescent signal to exceed background levels. Unlike microarray values, CT values are inversely proportional to the amount of target nucleic acid in a sample. A) qPCR expression of SLC25A20 pre- and post- cardioversion in the independent validation cohort. B) qPCR expression of PDK4. A symbol directly above a bar indicates a significant difference between groups; p <0.0005 (***), p <0.05(*). (TIFF) [file pone.0157550.s005.tiff]

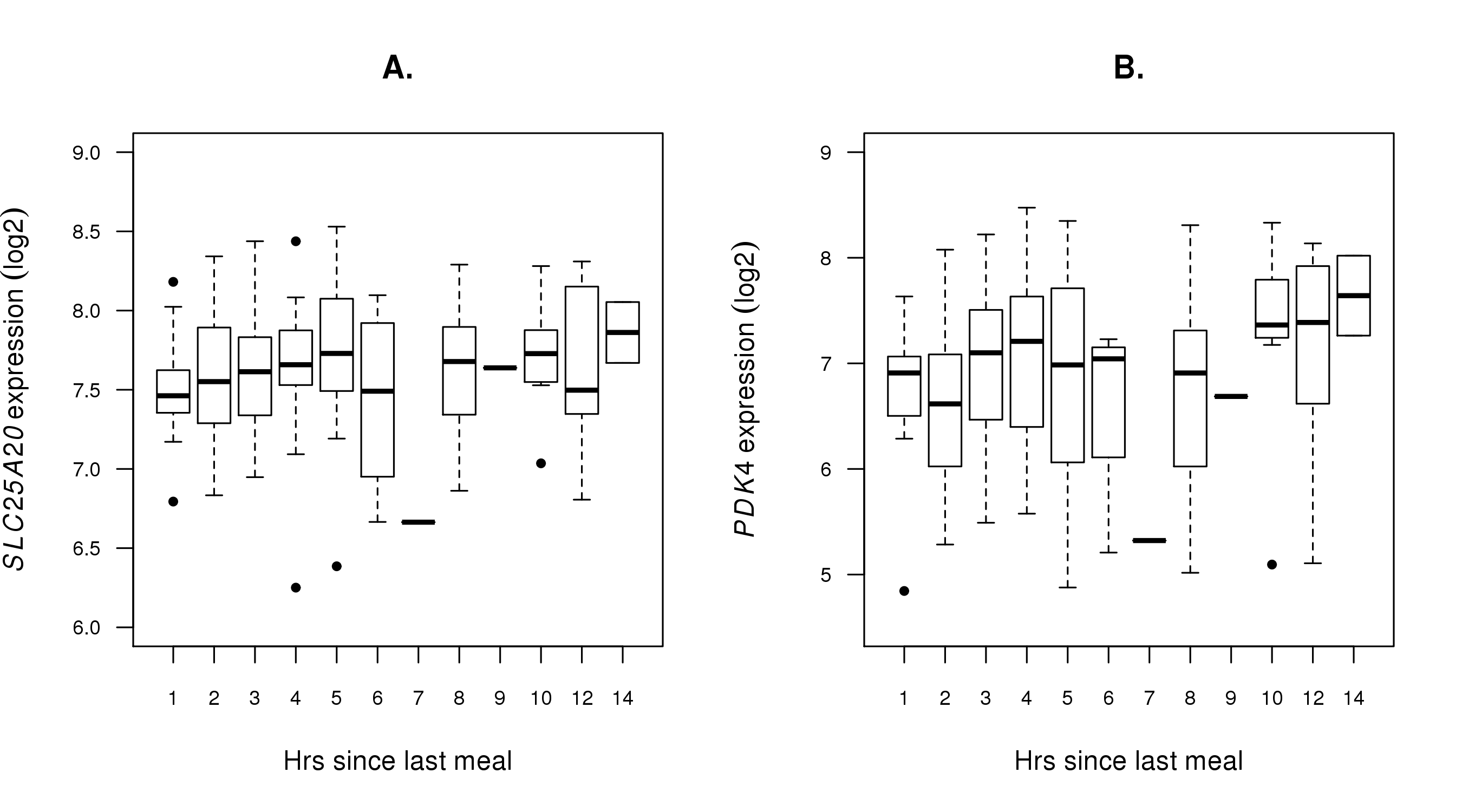

Supplement: S6 Fig — Boxes extend from the 25th to the 75th percentile, with the horizontal line representing the median. Outliers are identified as samples with an expression value 1.5 times more or less than the interquartile range. A) Expression of SLC25A20. B) Expression of PDK4. (TIFF) [file pone.0157550.s006.tiff]
