# Supplementary material for: Whole Blood Gene Expression Differentiates between Atrial Fibrillation and Sinus Rhythm after Cardioversion
Source: PLoS One. 2016 Jun 22;11(6):e0157550. doi: 10.1371/journal.pone.0157550 (PMC4917233; doi:10.1371/journal.pone.0157550)
Supplement: S2 Table — A logistic regression model was constructed for rhythm status. The model included NT-proBNP, SLC25A20, PDK4 and left atrial maximum volume. (DOCX) [file pone.0157550.s008.docx]

| Biomarker | Fold Change | Odds Ratio | P-value |
| --- | --- | --- | --- |
| *SLC25A20* | 7.1 | 17.0 | 0.0062 |
| *PDK4* | 1.8 | 2.4 | 0.05 |
| NT-proBNP | 1.0 | 1.0 | 0.0019 |
| Left atrial maximum volume | 0.99 | 0.99 | 0.48 |
