# Supplementary material for: Whole Blood Gene Expression Differentiates between Atrial Fibrillation and Sinus Rhythm after Cardioversion
Source: PLoS One. 2016 Jun 22;11(6):e0157550. doi: 10.1371/journal.pone.0157550 (PMC4917233; doi:10.1371/journal.pone.0157550)
Supplement: S3 Table — (DOCX) [file pone.0157550.s009.docx]

|  | Pre-cardioversion  (n=17) | Post-cardioversion  (n=17) | P-value |
| --- | --- | --- | --- |
| Gender, n (% female) | 6 (35.5) | 6 (35.3) |  |
| Age (years), mean ± SD | 68.7 ± 8.8 | 68.7 ± 8.8 |  |
| BMI (kg/m^2^), mean ± SD | 25.4 ± 4.2 | 25.5 ± 4.1 |  |
| Systolic BP (mmHg), mean ± SD | 138.7 ± 18.2 | 131.2 ± 10.7 | 0.087 |
| Diastolic BP (mmHg), mean ± SD | 84.4 ± 13.4 | 76.5 ± 7.7 | 0.029 |
|  |  |  |  |
| Medication, n(%) |  |  |  |
| Aspirin | 0 | 0 |  |
| Vitamin K Antagonist | 10 (58.8) | 10 (58.8) | 1 |
| Novel oral anticoagulants | 6 (35.5) | 6 (35.3) | 1 |
| Beta-blocker | 13 (76.5) | 12 (70.1) | 0.77 |
| Class III Antiarrhythmic | 6 (35.5) | 6 (35.5) | 1 |
| Class Ic Antiarrhythmic | 1 (5.9) | 2 (11.8) | 1 |
| ACE Inhibitor AT2 Antagonist | 12 (70.1) | 13 (76.5) | 1 |
| Diuretics | 7 (41.2) | 7 (41.2) | 1 |
| Calcium Antagonist | 0 | 0 |  |
| Digoxin | 2 (11.8) | 2 (11.8) | 1 |
|  |  |  |  |
| Heart rate (bpm) | 82.8 ± 13.3 | 57.4 ± 13.8 | 1.3x10^-5^ |
